# Fertility Intention Among Chinese Reproductive Couples During the COVID-19 Outbreak: A Cross-Sectional Study

**DOI:** 10.3389/fpubh.2022.903183

**Published:** 2022-06-21

**Authors:** Kun Chu, Ronghui Zhu, Yi Zhang, Wenjuan Pang, Xu Feng, Xiang Wang, Cheng Wu, Ningxia Sun, Wen Li

**Affiliations:** ^1^Center of Reproductive Medicine, Shanghai Key Laboratory of Embryo Original Diseases, International Peace Maternity and Child Health Hospital, School of Medicine, Shanghai Jiao Tong University, Shanghai, China; ^2^Department of Obstetrics and Gynecology, PLA Rocket Force Characteristic Medical Center, Beijing, China; ^3^Department of Military Health Statistics, Naval Medical University, Shanghai, China; ^4^Center of Reproductive Medicine, Second Affiliated Hospital of Naval Medical University, Shanghai, China

**Keywords:** COVID-19, fertility intention, reproductive couple, self-administered questionnaire, cross-sectional study

## Abstract

A decline in the fertility rate has been observed worldwide, which hampers social development severely. Given the impacts of COVID-19 on individuals and society, it is of great significance to investigate the fertility intention of reproductive couples under COVID-19. The convenience sampling method was used to obtain our study sample. The self-administered questionnaire included the following components: sociodemographic characteristics (age, residence, education, occupation, characteristics of the couples, and annual household income), reproductive history (parity, number of children, child gender, and duration of preparing pregnancy), and attitudes toward COVID-19, was distributed online *via* an applet of WeChat. The results showed that among 4,133 valid questionnaires, 1,091 had fertility intention before COVID-19, whereas 3,042 did not, indicating a fertility intention rate of 26.4% among participating couples. Of the 1,091 couples who had fertility intention before COVID-19, 520 (47.7%) were affected by the outbreak, whereas 571 (52.3%) were not. By multivariable logistic regression analysis, we further found that couples living in Hubei Province, the epicenter in China (OR 2.20, 95% CI 1.35–3.60), and couples who prepared for pregnancy longer before COVID-19 (OR 1.19, 95% CI 1.06–1.33) were more likely to change their fertility intention under the pandemic. In addition, most of the participants reported their fertility intention was affected by the inconvenience of seeking medical service under COVID-19. Therefore, more forms of medical services to provide convenience for patients might be effective ways to reverse the declined fertility intention rate in facing COVID-19.

## Introduction

Coronavirus disease 2019 (COVID-19) is a highly infectious disease, the rampant spread of which has been declared by the WHO as a global public health emergency (1, 2). In December 2019, COVID-19 was first reported in Wuhan, Hubei Province, China, followed by an outbreak across the country and the world (1, 2). By 1 May 2022, over 513.4 million COVID-19 cases and over 6.3 million deaths have been reported worldwide (3). Apart from threatening human life and health, COVID-19 also brings economic burden as well as various psychological distresses, such as panic disorders, anxiety, and depression (4–6). Fighting against COVID-19 is still a global priority.

A decline in the fertility rate has been observed worldwide along with modernization, as a result of delayed marriage, increased employment, higher education levels, and so on (7–10). In China, despite the relaxation of the one-child policy in 2013 and the implementation of the two-child policy in 2016, the annual birth number has not exhibited an obvious growth (11). Though aging populations are increasing worldwide, this process is extremely accelerated in China, as a result of the one-child policy (11). The aging process together with decreased fertility rate will result in a shrinking workforce and growing health expenditure, which hampers economic and social development (12, 13). A three-child policy has just been released on May 31, 2021, in China, however, the third birth intention of the childbearing-age population in China is still low after the announcement (14). Reversing declined fertility is still a primary concern around the world.

Numerous studies have shown that public health emergencies can not only bring psychological stress but also change individual behavior (6). In facing public health emergencies, such as the Zika virus, pregnancy postponement and a decline in live births were observed (15, 16), indicating an impact of public health emergencies on individual fertility behavior. Also, a cross-sectional study based on fertility patients under COVID-19 showed that about 28% of the patients did not wish to renew their fertility treatments. The main reason was the concern of being infected, followed by financial concerns, risks to the pregnancy, and fear of delivering an infant in the current situation (17).

Fertility intention has been defined in a few ways based on fertility desires, attitudes, and behaviors (18). In the present study, we focus on the fertility desire to have children in consideration of various limitations, like number of children, gender, the interval between pregnancies, age, work, and so on. It is reported that women's fertility intentions are related to both macro- and micro-level factors, such as social-cultural environment, family preference, and personal characteristics (18, 19). In spite of the discrepancy between fertility intention and the actual birth, the latter can be predicted by the former to some extent (20–22). Since the relationship between fertility intention and COVID-19 is still under-researched, it is of great significance to investigate its influence on the fertility intention of reproductive couples. Therefore, the current study investigated the fertility intention among Chinese reproductive couples during the COVID-19 outbreak and their concerns, which would provide new evidence for how to manage reproductive couples in facing COVID-19.

## Methods

### Participants

This is a cross-sectional study by means of a self-administered questionnaire. A non-probability sampling method, the convenience sampling method, was used to obtain our sample. The investigators included in the sample were from Wuhan, Shanghai, Guangzhou, Hangzhou, and other cities.

The anonymous questionnaire was designed and reviewed by both clinicians and statisticians, which included the following components: sociodemographic characteristics (age, residence, education, occupation, characteristics of the couples, and annual household income), reproductive history (parity, number of children, child gender, and duration of preparing pregnancy), and attitudes toward COVID-19. The English version of the full questionnaire is enclosed as a Supplementary Table 1.

The questionnaires were distributed during 20–30 April 2020, when the pandemic was relatively under control in China. On account of the outbreak of the COVID-19, the Chinese government advised the public to avoid face-to-face interactions. Therefore, this survey was conducted online *via* WeChat, one of the most popular social media platforms with a prevalence of more than 90% in major cities in China. In order to control the data quality well, we specially developed a WeChat applet for investigation. Two distribution patterns, one-by-one WeChat messages and advertising on our WeChat official accounts, were used. Couples of reproductive age (20–50 y) were included. Notably, each eligible couple could only fill out the questionnaire once, and only completed questionnaires could be submitted online.

This study was approved by the Ethics Committee of Second Affiliated Hospital of Naval Medical University and electronic informed consent was obtained before filling in the questionnaire.

### Statistical Analysis

All analyses were conducted with SAS 9.4 (SAS Institute Inc, Cary, NC). All reported probability values were 2-tailed, and the criterion for significance was set at *p* = 0.05 unless otherwise stated. We reported a confidence interval at a significance level of 0.05.

Since variables of our study were all categorical or ranked variables, data were expressed as frequency and percentage. In the univariate analysis, the chi-square test was used for categorical variables, and the Cochran-Mantel-Haenszel tests were applied to ranked variables. We investigated the risk for fertility intention using multivariable logistic regression models, and calculated the odds ratio (OR) and corresponding 95% confidence interval (CI). Variables with a level of statistical significance of 0.2 or less in the univariate analysis and variables which were considered meaningful professionally were entered into a logistic regression model. We constructed two models, one of which used the full entry method and the other used the stepwise selection method (α_enter_ = 0.10, α_remove_ = 0.15). A Forest plot was also provided for the models.

We performed a subgroup analysis to investigate the risk for fertility intention in different groups of people using multivariable logistic regression models. We did not impute data due to no missing data.

## Results

A total of 4,133 valid questionnaires were received, of which 1,091 couples had fertility intention before COVID-19, whereas 3,042 did not, indicating a fertility intention rate of 26.4% among the participating couples. The baseline characteristics of the participants were shown in Table 1. Consisting with the former investigations, multivariable logistic regression analysis showed that age, residence, occupation, occupation of spouse, marital status, parity, number of children, and annual household income were the significant factors associated with fertility intention as shown in Supplementary Table 2 (18, 19). In addition, we found that the number of living parents could also influence fertility intention. Couples with more living parents (OR 1.17, 95% CI 1.04–1.32) were more likely to have fertility intention.

**Table 1:** Basic information of 4,133 respondents.

	**Group 1** **(*N* = 1,091)**	**Group 2** **(*N* = 3,042)**	**Statistic**	***p*** **-value**
**Age of women**			177.5865	<0.0001
≤30 y	338 (31.0)	613 (20.2)		
31–35 y	453 (41.5)	911 (29.9)		
36–40 y	196 (18.0)	752 (24.7)		
41–45 y	84 (7.7)	499 (16.4)		
≥45 y	20 (1.8)	267 (8.8)		
**Age of men**			179.2270	<0.0001
≤30 y	275 (25.2)	518 (17.0)		
31–35 y	455 (41.7)	818 (26.9)		
36–40 y	210 (19.2)	707 (23.2)		
41–45 y	103 (9.4)	564 (18.5)		
≥45 y	48 (4.4)	435 (14.3)		
**Residence**			61.6688	<0.0001
Shanghai City	441 (40.4)	1,644 (54.0)		
Hubei Province	89 (8.2)	219 (7.2)		
Henan Province	26 (2.4)	49 (1.6)		
Zhejiang Province	162 (14.8)	328 (10.8)		
Guangdong Province	21 (1.9)	44 (1.4)		
Other	352 (32.3)	758 (24.9)		
**Education**			10.1449	0.0014
Primary school or less	6 (0.5)	6 (0.2)		
Middle school	69 (6.3)	146 (4.8)		
High school	109 (10.0)	493 (16.2)		
Junior College	240 (22.0)	713 (23.4)		
University	486 (44.5)	1,307 (43.0)		
Advanced degree	181 (16.6)	377 (12.4)		
**Occupation of women**			42.7267	<0.0001
Farmer	16 (1.5)	25 (0.8)		
Worker	22 (2.0)	97 (3.2)		
Civil servant	46 (4.2)	77 (2.5)		
Professional & technical	296 (27.1)	887 (29.2)		
Office worker	392 (35.9)	1,094 (36.0)		
Self-employed	91 (8.3)	168 (5.5)		
Unemployed	46 (4.2)	70 (2.3)		
Other	182 (16.7)	624 (20.5)		
**Occupation of men**			21.6570	0.0029
Farmer	15 (1.4)	25 (0.8)		
Worker	32 (2.9)	136 (4.5)		
Civil servant	80 (7.3)	235 (7.7)		
Professional and technical	210 (19.2)	678 (22.3)		
Office worker	397 (36.4)	1,117 (36.7)		
Self-employed	151 (13.8)	306 (10.1)		
Unemployed	15 (1.4)	41 (1.3)		
Other	191 (17.5)	504 (16.6)		
**Marital status**			11.9048	0.0006
First marriage	1,021 (93.6)	2,924 (96.1)		
Remarriage	70 (6.4)	118 (3.9)		
**Parity**			556.0452	<0.0001
0	641 (58.8)	519 (17.1)		
1	385 (35.3)	1,906 (62.7)		
2	59 (5.4)	588 (19.3)		
3 or above	6 (0.5)	29 (1.0)		
**Number of children**			526.6226	<0.0001
0	620 (56.8)	478 (15.7)		
1	394 (36.1)	1,908 (62.7)		
2	66 (6.0)	603 (19.8)		
3 or above	11 (1.0)	53 (1.7)		
**Child gender**			17.1129	0.0002
Male	165 (35.0)	989 (38.6)		
Female	263 (55.8)	1,198 (46.7)		
Male and Female	43 (9.1)	377 (14.7)		
**Annual household income**			0.8791	0.3484
<¥100,000	199 (18.2)	534 (17.6)		
¥100,000–150,000	283 (25.9)	750 (24.7)		
¥150,000–200,000	193 (17.7)	564 (18.5)		
>¥200,000	416 (38.1)	1,194 (39.3)		
**Number of parents alive**			43.7300	<0.0001
0	6 (0.5)	31 (1.0)		
1	9 (0.8)	89 (2.9)		
2	55 (5.0)	273 (9.0)		
3	185 (17.0)	603 (19.8)		
4	836 (76.6)	2,046 (67.3)		
**Duration of preparing pregnancy before COVID-19**			-	-
Not started	363 (33.3)	-		
<1 y	300 (27.5)	-		
1–2 y	217 (19.9)	-		
>3 y	211 (19.3)	-		

*Data are expressed as n (%)*.

*Group 1: participants with fertility intention before COVID-19; Group 2: participants without fertility intention before COVID-19*.

*Dichotomous or nominal categorical variables were analyzed using the Pearson chi-square test; Ordinal categorical variables were analyzed using the Cochran-Mantel-Haenszel test*.

Of the 1,091 couples who had fertility intention before COVID-19, 520 (47.7%) were affected by the outbreak, whereas 571 (52.3%) were not. The baseline characteristics of these two groups are shown in Table 2. About 60% of couples under 35 years old who had fertility intention before COVID-19 were affected by the outbreak, about 17% of couples aged 36–40 were affected, and about 10% of couples over the age of 40 were affected. To find the factors which can influence the fertility intention during the COVID-19, a multivariable logistic regression analysis was conducted. The results showed that residence and duration of preparing for pregnancy before COVID-19 were the significant factors associated with changed fertility intention. Couples living in Hubei Province, the hardest-hit area of the epidemic (OR 2.20, 95% CI 1.35–3.60), and couples who prepared for pregnancy longer before COVID-19 (OR 1.19, 95% CI 1.06–1.33) were more likely to change their fertility intention. Other factors, such as education level, marital status, parity, children number, and annual household income, were not significantly correlated with changes in fertility intention (Table 3).

**Table 2:** Basic information of 1,091 respondents with fertility intention before COVID-19.

	**Group 1** **(*N* = 520)**	**Group 2** **(*N* = 571)**	**Statistic**	***p*** **-value**
**Age of women**			0.0152	0.9019
≤30 y	158 (30.4)	180 (31.5)		
31–35 y	224 (43.1)	229 (40.1)		
36–40 y	89 (17.1)	107 (18.7)		
41–45 y	39 (7.5)	45 (7.9)		
≥45 y	10 (1.9)	10 (1.8)		
**Age of men**			0.0043	0.9477
≤30 y	120 (23.1)	155 (27.1)		
31–35 y	236 (45.4)	219 (38.4)		
36–40 y	92 (17.7)	118 (20.7)		
41–45 y	51 (9.8)	52 (9.1)		
≥45 y	21 (4.0)	27 (4.7)		
**Residence**			16.5801	0.0054
Shanghai City	193 (37.1)	248 (43.4)		
Hubei Province	59 (11.3)	30 (5.3)		
Henan Province	13 (2.5)	13 (2.3)		
Zhejiang Province	71 (13.7)	91 (15.9)		
Guangdong Province	11 (2.1)	10 (1.8)		
Other	173 (33.3)	179 (31.3)		
**Education**			1.4327	0.2313
Primary school or less	4 (0.8)	2 (0.4)		
Middle school	36 (6.9)	33 (5.8)		
High school	55 (10.6)	54 (9.5)		
Junior College	112 (21.5)	128 (22.4)		
University	231 (44.4)	255 (44.7)		
Advanced degree	82 (15.8)	99 (17.3)		
**Occupation of women**			6.4258	0.4910
Farmer	8 (1.5)	8 (1.4)		
Worker	14 (2.7)	8 (1.4)		
Civil servant	28 (5.4)	18 (3.2)		
Professional and technical	135 (26.0)	161 (28.2)		
Office worker	186 (35.8)	206 (36.1)		
Self-employed	41 (7.9)	50 (8.8)		
Unemployed	23 (4.4)	23 (4.0)		
Other	85 (16.3)	97 (17.0)		
**Occupation of men**			2.7577	0.9065
Farmer	6 (1.2)	9 (1.6)		
Worker	15 (2.9)	17 (3.0)		
Civil servant	43 (8.3)	37 (6.5)		
Professional and technical	98 (18.8)	112 (19.6)		
Office worker	185 (35.6)	212 (37.1)		
Self-employed	69 (13.3)	82 (14.4)		
Unemployed	8 (1.5)	7 (1.2)		
Other	96 (18.5)	95 (16.6)		
**Marital status**			1.1654	0.2804
First marriage	491 (94.4)	530 (92.8)		
Remarriage	29 (5.6)	41 (7.2)		
**Parity**			1.6679	0.1965
0	315 (60.6)	326 (57.1)		
1	178 (34.2)	207 (36.3)		
2	24 (4.6)	35 (6.1)		
3 or above	3 (0.6)	3 (0.5)		
**Number of children**			1.1128	0.2915
0	303 (58.3)	317 (55.5)		
1	183 (35.2)	211 (37.0)		
2	30 (5.8)	36 (6.3)		
3 or above	4 (0.8)	7 (1.2)		
**Child gender**			0.3739	0.8295
Male	79 (36.4)	86 (33.9)		
Female	118 (54.4)	145 (57.1)		
Male and Female	20 (9.2)	23 (9.1)		
**Annual household income**			3.5667	0.0589
< ¥100,000	100 (19.2)	99 (17.3)		
¥100,000–150,000	151 (29.0)	132 (23.1)		
¥150,000–200,000	80 (15.4)	113 (19.8)		
>¥200,000	189 (36.3)	227 (39.8)		
**Number of parents alive**			0.0310	0.8602
0	3 (0.6)	3 (0.5)		
1	3 (0.6)	6 (1.1)		
2	23 (4.4)	32 (5.6)		
3	96 (18.5)	89 (15.6)		
4	395 (76.0)	441 (77.2)		
**Duration of preparing pregnancy before COVID-19**			7.2325	0.0072
Not started	162 (31.2)	201 (35.2)		
<1 y	130 (25.0)	170 (29.8)		
1–2 y	113 (21.7)	104 (18.2)		
>3 y	115 (22.1)	96 (16.8)		

*Data are expressed as n (%)*.

*Group 1: participants with fertility intentions affected after the COVID-19 outbreak; Group 2: participants with fertility intentions unaffected after the COVID-19 outbreak*.

*Dichotomous or nominal categorical variables were analyzed using the Pearson chi-square test; Ordinal categorical variables were analyzed using the Cochran-Mantel-Haenszel test*.

**Table 3:** Multivariable logistic regression analysis (respondents with fertility intention before COVID-19).

**Variable^a^**	**Model 1^b^**		**Model 2^b^**	
	**OR (95% CI)**	***P*** **-value**	**OR (95% CI)**	***p*** **-value**
**Residence**		0.0042		0.0031
Other	Reference		Reference	
Shanghai City	0.86 (0.65,1.16)		0.84 (0.63,1.12)	
Hubei Province	2.24 (1.37,3.67)		2.20 (1.35,3.60)	
Henan Province	1.06 (0.48,2.38)		1.06 (0.48,2.37)	
Zhejiang Province	0.78 (0.53,1.14)		0.76 (0.52,1.11)	
Guangdong Province	1.28 (0.53,3.14)		1.22 (0.50,2.97)	
Education	0.97 (0.86,1.11)	0.6915		
**Marital status**		0.1771		
First marriage	Reference			
Remarriage	0.70 (0.42,1.17)			
Parity	0.96 (0.69,1.35)	0.8217		
Number of children	0.97 (0.71,1.32)	0.8287		
Annual household income	0.95 (0.84,1.07)	0.3711		
Duration of preparing pregnancy before COVID-19	1.17 (1.04,1.32)	0.0073	1.19 (1.06,1.33)	0.0024

a *Include variables with p-value <0.2 in the univariable analysis, as well as education, marital status, and number of children*.

b *Model 1 is the complete model which includes all variables, Model 2 is the selected model using a stepwise selection method (α_enter_ = 0.10, α_remove_ = 0.15) to select variables*.

*OR, odds ratio; CI, confidence interval*.

**Figure 1:**
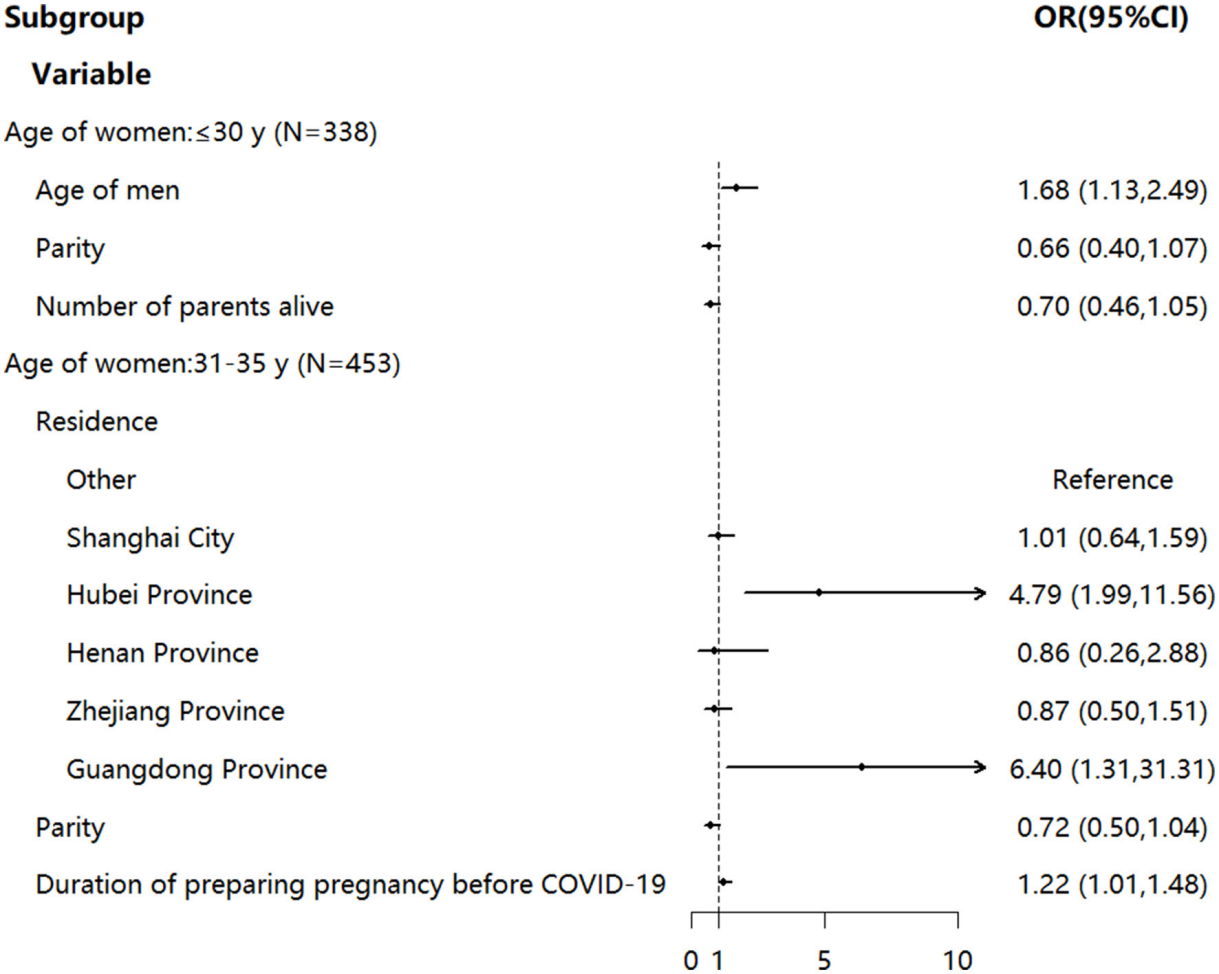
Subgroup multivariable logistic regression analysis (respondents with fertility intention before COVID-19). OR was indicated by an arrow if the value exceeded 10. OR, odds ratio; CI, confidence interval.

Subgroup multivariable logistic regression analyses were further conducted (Figure 1). In the group of women under 30 years old, the age of their spouses, parity, and number of living parents were the significant factors associated with changed fertility intention. Women whose spouse was older were more likely to change fertility intention (OR 1.68, 95% CI 1.13–2.49). Those who had delivered fewer liveborn children might be more likely to change their fertility intention (OR 0.66, 95% CI 0.40–1.07). In addition, women with fewer living parents might be more likely to change their fertility intention (OR 0.70, 95% CI 0.46–1.05). As for the women of 31–35 years old, residence, parity, and duration of preparing pregnancy before COVID-19 were significant factors associated with changed fertility intention. Women living in Guangdong Province (OR 6.40, 95% CI 1.31–31.31) and Hubei Province (OR 4.79, 95% CI 1.99–11.56) were more likely to change their fertility intention than others. Also, women who prepared for pregnancy for a longer duration before COVID-19 were more likely to change their fertility intention (OR 1.22, 95% CI 1.01–1.48). Consistent with the group under 30 years old, those who had delivered fewer liveborn children might be more likely to change their fertility intention (OR 0.72, 95% CI 0.50–1.04). However, no significant factors were found in groups over 35 years old.

We also investigated the participants' attitudes toward COVID-19. As shown in Table 4, most of the couples (38.0%) changed their fertility intention because of the inconvenience of seeking medical service during the pandemic; about 9.3% worried about infection risk during pregnancy; 8.6% changed because of the economic burden caused by COVID-19; 7.2% worried about the risk of COVID-19 on fetal development. Though the situation was improving in China, only 26.1% of the participants had fertility intention at that time, while 17.4% of the participants had fertility intention before COVID-19, and 94.2% of those who did not, reported that they did not want to have children at that time. Among participants who had fertility intention after the situation improved, 62.8% would start to prepare for pregnancy right then, 16.0% would like to wait for a better situation in China, 5.4% would like to wait for a better situation worldwide, and 15.9% decided to wait for the end of the pandemic. We found 63.0% reported their willingness to see doctors was hindered on account of COVID-19, while 37.0% were not. Compared with the portion with immediate fertility intention (26.1%), more couples (30.4%) expressed their willingness to get pregnant after COVID-19 is completely over.

**Table 4:** Attitudes toward COVID-19 of participants with fertility intention before COVID-19.

**Does COVID-19 change your fertility intention?**	
Yes	520 (47.7%)
No	571 (52.3%)
**Why does COVID-19 change your fertility intention?**	
Risk of COVID-19 infection during pregnancy	102 (9.3%)
Risk of COVID-19 on fetal development	79 (7.2%)
Inconvenience of seeking medical service during the COVID-19	415 (38.0%)
Economic burden caused by COVID-19	94 (8.6%)
Potential work stress after COVID-19 is over	70 (6.4%)
Short of hands during pregnancy and postpartum if family member is infected	38 (3.5%)
Others	293 (26.9%)
**Since the situation is getting better in China, do you have fertility intention right now?**	
Yes	1,078 (26.1%)
No	3,055 (73.9%)
**If you have fertility intention right now, when will you start preparing pregnancy?**	
Right now	677 (62.8%)
Waiting for better situation in China	172 (16.0%)
Waiting for better situation worldwide	58 (5.4%)
Waiting COVID-19 completely over	171 (15.9%)
**Which of the following measures will you take if you prepare pregnancy?**	
None	299 (27.7%)
Predicting ovulation by tracking menstrual cycle or software	202 (18.7%)
Pre-pregnancy physical examination and guidance from doctor	191 (17.7%)
Assistance of assisted reproductive medicine	352 (32.7%)
Others	34 (3.2%)
**Does COVID-19 impact your willingness to see doctors?**	
Yes	679 (63.0%)
No	399 (37.0%)
**Are you going to get pregnant after COVID-19 is over?**	
Yes	1,257 (30.4%)
No	2,876 (69.6%)
**Which of the following knowledge or assistance is upmost to your family?**	
None	1,692 (40.9%)
The influence of COVID-19 on pregnancy	464 (11.2%)
Basic knowledge of reproductive health and pregnancy	397 (9.6%)
Knowledge and assistance of assisted reproductive medicine	296 (7.2%)
Policies and regulations of COVID-19 issued by the government	912 (22.1%)
Others	372 (9.0%)

*Data are expressed as n (%)*.

## Discussion

In the present study, we investigated the fertility intention of reproductive couples under COVID-19 in China and their attitudes toward the disease. The 4,133 valid questionnaires indicated a fertility intention rate of 26.4% among the participated couples. Of the 1,091 couples who had fertility intention before COVID-19, 47.7% were affected by the outbreak. By multivariable logistic regression analysis, we found that residence and duration of preparing pregnancy before COVID-19 were the significant factors associated with changed fertility intention. Couples living in Hubei Province, the epicenter in China, and couples with a longer duration of preparing for pregnancy before COVID-19 were more likely to change their fertility intention.

Fertility intention is comprised of tempo intentions, namely the timing of childbirth, and quantum intentions, namely the total number of children (19). Several studies have confirmed its role as a reliable birth predictor when taking into account demographic and socioeconomic factors (22). Both macro and micro level factors can influence fertility intention (23–27). Our results showed that couples living in Hubei Province, the epicenter in China, and couples who spent longer preparing for pregnancy before COVID-19 were more likely to change their fertility intention. Given that most couples changed their fertility intention because of the inconvenience of seeking medical service during the pandemic, it can be easily explained why couples living in Hubei Province were more likely to change their fertility intention. Couples who prepared for pregnancy longer before COVID-19 being more likely to change their fertility intention might result from greater cautiousness of the couples who prepared for pregnancy longer.

Parity and number of living parents were potential associated factors based on the subgroup analysis results. It might be because that women who delivered fewer liveborn children had less pregnancy and delivery experience than those who delivered more liveborn children, so the former might be more likely to change their fertility intention for fear and stress of the unknown. As for the number of parents alive, it might be because more living parents could provide more help during pregnancy and postpartum period under COVID-19. Given the association of economic status with the fertility intention as well as the potential economic burden under the pandemic, we have assumed that economic status might be an associated factor of changed fertility intention. Contrary to our expectations, annual house income, namely the economic status of the couples, did not exhibit any correlation with changed fertility intention either in the overall analysis or the subgroup analysis. This might be due to the rapid recovery of China under COVID-19 as well as the intrinsic, thrifty, money-saving habits of the Chinese people.

As the Chinese government rapidly took effective actions after the COVID-19 outbreak, the pandemic was relatively under control in China at the time of this survey. Although the situation was getting better in China, only 26.1% of the participants had fertility intention at that time, among which 62.8% would start to prepare for pregnancy right then, 16.0% would like to wait for a better situation in China, 5.4% would like to wait for better situation worldwide, and 15.9% decided to wait for the end of the pandemic. This indicated that the pandemic not only impacted the fertility intentions of the reproductive couples but also postponed the process. A majority, 63.0%, of the participants reported that their willingness to see doctors was hindered on account of COVID-19, while 37.0% were not, and this was consistent with the fact that most of the couples (38.0%) changed fertility intention because of the inconvenience of seeking medical service during the pandemic. Easily access to medical services may reverse the decreased fertility intention rate in facing COVID-19. Clinicians are suggested to provide more forms of online services in order to provide convenience for the patients.

The present study is subject to a number of limitations. First of all, due to the cross-sectional design, the results we found are unable to be defined as causal. Also, the self-report design may lead to random selection by the participants. However, we do not think this can be an important source of bias since we have controlled the number of the questions. In addition, the number of participants was not completely balanced across the cities, which may introduce bias to the study. In future investigations, multi-center research would be conducted to avoid bias. Nevertheless, analysis of cross-sectional data proves very useful and can generate hypotheses to be tested by future prospective studies.

The strength of our study was that the sample size is large; the cities covered are representative, such as the geographical location, the level of economic development, the culture, and the severity of COVID-19; and our self-developed network survey tool is high-quality. The respondents finished the questionnaire on the WeChat platform. Only the respondents who were authorized to log in can access the questionnaire. After authorization, the basic information about the respondents' WeChat can be obtained. Through the unique identification in the data, the corresponding WeChat account of the respondent can be found to ensure authenticity and reliability. The dataset was saved on a cloud server to detect the intrusion information in real-time. The interface of our WeChat small program for data collection used HTTPS protocol to ensure that the data will not be tampered with during transmission and the data integrity was guaranteed.

## Conclusion

We reported a fertility intention rate of 26.4% among the Chinese reproductive couples. Under COVID-19, about 47.7% changed their fertility intention, whereas 52.3% did not. Further analysis showed that couples living in the epicenter in China, and couples with a longer duration of preparing for pregnancy before COVID-19 were more likely to change their fertility intention. Most of the participants reported their fertility intention was affected by the inconvenience of seeking medical service during the pandemic. More forms of medical services in order to provide convenience for the patients might be effective ways to reverse the declined fertility intention rate in facing COVID-19.

## Data Availability Statement

The original contributions presented in the study are included in the article/Supplementary Material, further inquiries can be directed to the corresponding authors.

## Ethics Statement

The studies involving human participants were reviewed and approved by Ethics Committee of Second Affiliated Hospital of Naval Medical University. The participants provided their electronic informed consent to participate in this study.

## Author Contributions

KC, RZ, and YZ contributed equally to this work, authored this manuscript, and performed the research. WL, NS, and CW contributed conception of the study, designed the study, prepared the tables and figures, and revised the manuscript. KC, RZ, YZ, WP, XF, and XW performed the statistical analysis and interpretation. KC wrote the first draft of the manuscript. All authors contributed to the article and approved the submitted version.

## Funding

This study was supported by the Shanghai Hospital Development Center Major Clinical Research Project (SHDC2020CR3054B), National Natural Science Foundation of China (82071605, 81873821, and 81901482), Military Innovation Project Special Project (18JS009), Science and Technology Commission of Shanghai Municipality Medical Guidance Project (19411960700), and Shanghai Municipal Health and Family Planning Commission Chinese Medicine Research Special Project (2016JP009).

## Conflict of Interest

The authors declare that the research was conducted in the absence of any commercial or financial relationships that could be construed as a potential conflict of interest. The reviewer LZ declared a shared affiliation with the author(s) KC and WL to the handling editor at the time of review.

## Publisher's Note

All claims expressed in this article are solely those of the authors and do not necessarily represent those of their affiliated organizations, or those of the publisher, the editors and the reviewers. Any product that may be evaluated in this article, or claim that may be made by its manufacturer, is not guaranteed or endorsed by the publisher.
