# Supplementary material for: Fertility Intention Among Chinese Reproductive Couples During the COVID-19 Outbreak: A Cross-Sectional Study
Source: Front Public Health. 2022 Jun 21;10:903183. doi: 10.3389/fpubh.2022.903183 (PMC9253424; doi:10.3389/fpubh.2022.903183)
Supplement: Supplementary file 1 [file Data_Sheet_1.pdf]

Supplementary Material

Supplementary Table

TABLE S1 The English version of the full questionnaire.

**1. Age of women**
≤30 y
31-35 y
36-40 y
41-45 y
≥45 y
**2. Age of men**
≤30 y
31-35 y
36-40 y
41-45 y
≥45 y
**3. Residence**
Shanghai City
Hubei Province
Henan Province
Zhejiang Province
Guangdong Province
Other
**4. Education**
Primary school or less
Middle school
High school
Junior College
University
Advanced degree
**5. Occupation of women**
Farmer
Worker
Civil servant
Professional & technical
Office worker
Self-employed
Unemployed
Other
**6. Occupation of men**
Farmer
Worker
Civil servant
Professional & technical
Office worker
Self-employed
Unemployed
Other
**7. Marital status**

TABLE S1 cont. The English version of the full questionnaire.

First marriage
Remarriage
**8. Parity**
0
1
2
3 or above
**9. Number of children**
0
1
2
3 or above
**10. Child gender**
Male
Female
Male & Female
**11. Annual household income**
<¥100,000
¥100,000-150,000
¥150,000-200,000
>¥200,000
**12. Number of parents alive**
0
1
2
3
4
**13. Do you have fertility intention before COVID-19**
Yes
No
**14. If you have fertility intention before COVID-19, how long have you prepared before COVID-19**
Not started
<1y
1-2y
>3y
**15. Does COVID-19 change your fertility intention?**
Yes
No
**16. Why does COVID-19 change your fertility intention?**
Risk of COVID-19 infection during pregnancy
Risk of COVID-19 on fetal development
Inconvenience of seeking medical service during the COVID-19
Economic burden caused by COVID-19
Potential work stress after COVID-19 is over
Short of hands during pregnancy and postpartum if family member is infected
Others
**17. Since the situation is getting better in China, do you have fertility intention right now?**
Yes
No
**18. If you have fertility intention right now, when will you start preparing pregnancy?**
Right now

TABLE S1 cont. The English version of the full questionnaire.
Waiting for better situation in China
Waiting for better situation worldwide
Waiting COVID-19 completely over
**19. Which of the following measures will you take if you prepare pregnancy?**
None
Predicting ovulation by tracking menstrual cycle or software
Pre-pregnancy physical examination and guidance from doctor
Assistance of assisted reproductive medicine
Others
**20. Does COVID-19 impact your willingness to see doctors?**
Yes
No
**21. Are you going to get pregnant after COVID-19 is over?**
Yes
No
**22. Which of the following knowledge or assistance is upmost to your family?**
None
The influence of COVID-19 on pregnancy
Basic knowledge of reproductive health and pregnancy
Knowledge and assistance of assisted reproductive medicine
Policies and regulations of COVID-19 issued by the government
Others

TABLE S2 Multivariable logistic regression analysis (all participants).

Variable[a]	Model[b]	
	OR(95%CI)	*p* value
Age of women	0.91 (0.84,0.99)	0.0259
Residence		<.0001
Other	Reference	
Shanghai City	0.47 (0.39,0.57)	
Hubei Province	0.97 (0.71,1.33)	
Henan Province	1.46 (0.83,2.57)	
Zhejiang Province	1.13 (0.87,1.46)	
Guangdong Province	1.16 (0.61,2.17)	
Occupation of women		0.0118
Other	Reference	
Farmer	3.19 (0.94,10.82)	
Worker	1.29 (0.68,2.45)	
Civil servant	1.65 (1.02,2.67)	
Professional & technical	1.19 (0.91,1.55)	
Office worker	1.10 (0.85,1.42)	
Self-employed	1.60 (1.08,2.39)	
Unemployed	2.14 (1.31,3.48)	
Occupation of men		0.0434
Other	Reference	
Farmer	1.45 (0.41,5.10)	
Worker	0.96 (0.55,1.66)	
Civil servant	0.89 (0.61,1.28)	
Professional & technical	0.96 (0.72,1.29)	
Office worker	1.09 (0.84,1.40)	
Self-employed	1.62 (1.16,2.26)	
Unemployed	1.65 (0.80,3.41)	
Marital status		<.0001
First marriage	Reference	
Remarriage	2.80 (1.96,4.01)	
Parity	0.35 (0.27,0.44)	<.0001
Number of children	0.56 (0.45,0.70)	<.0001
Annual household income	1.09 (1.01,1.18)	0.0206
Number of surviving parents	1.17 (1.04,1.32)	0.0075

Note: a, Include all variables except Child gender in Table 1.
b, selected model using a stepwise selection method (α_{enter}=0.10, α_{remove}=0.15) to select variables.
OR, odds ratio; CI, confidence interval
